# Mating success and potential male-worker conflict in a male-dimorphic ant

**DOI:** 10.1186/1471-2148-7-114

**Published:** 2007-07-10

**Authors:** Alexandra Schrempf, Eric Darrouzet, Jürgen Heinze

**Affiliations:** 1 Biologie I, Universität Regensburg, Universitätsstraße 31, D-93040, Regensburg, Germany; 2 Université de Tours, Institut de Recherche sur la Biologie de l'Insecte, IRBI UMR CNRS 6035, parc de Grandmont, 37200 Tours, France

## Abstract

**Background:**

Males of many species adjust their reproductive tactics with regard to their condition and status. For example, large males may develop weapons and fight for access to females, whereas small or undernourished males do not express costly weapons or ornaments and sneak copulations. Different condition-dependent reproductive tactics may be associated with unequal average fitness, but the tactic chosen by a given male under given circumstances is thought to result in the highest possible fitness return.

The ant species *Cardiocondyla obscurior *exhibits an environment-controlled polymorphism of docile, winged males and aggressive "ergatoid" males. Ergatoid males, which can replenish their sperm supply throughout their lives, engage in lethal fighting, and attempt to monopolize all female sexuals available in their nests, were previously assumed to gain higher lifetime reproductive success than the peaceful, winged males, which disperse to mate away from the nest and whose spermatogenesis is limited to the first days of adult life. However, precise data on male mating success have as yet not been available.

Here, we compare the average mating success of the two male morphs, taking the high mortality rate of immature ergatoid males into account. Because individuals in insect societies may have opposing interests about their own development, we also investigate whether the interests of male larvae coincide with those of the workers and the rest of the society.

**Results:**

When the survival probability of males is taken into account, winged males are more likely to mate multiply and in consequence have a higher estimated average mating success than ergatoid males. Therefore, male larvae are expected to prefer developing into winged instead of ergatoid adults.

**Conclusion:**

Though male larvae can expect a higher average mating success when developing into winged males, most colonies produce only ergatoid males under standard conditions. This might point at a novel type of potential kin conflict within the social insect colony. Because workers in insect societies usually control male larval development, ergatoid male production under normal conditions probably reflects the optimal allocation strategy of workers to maximise their inclusive fitness.

## Background

Males of many animal species have evolved different ways of increasing their reproductive success. Such "alternative reproductive tactics" are often associated with differences in body size or different expression of morphological traits, such as weapons or ornaments, which again are controlled either by a genetic polymorphism or the environment during a certain life stage. Environment-based polymorphisms can be maintained in a population by status-dependent selection [1-3] i.e., an individual's condition determines which reproductive tactic (e.g., fighting vs. sneaking) it adopts [1]. Generally, individuals appear to choose the tactic that yields the highest fitness with regard to their competitive ability, and individuals therefore are often considered doing the "best of a bad job" [4].

Males of the ant *Cardiocondyla obscurior *(Wheeler, 1929) exhibit an environmentally determined fighter-flier polymorphism with wingless "ergatoid" males and winged males. The two male morphs differ in various morphological, physiological, behavioural and life history traits. Ergatoid males are robust, have small eyes and long, sabre-shaped mandibles [5]. They are produced year-round and usually stay in their natal nests, where they engage in lethal fighting with other ergatoid males. Older ergatoid males use their mandibles to puncture the not yet sclerotised cuticle of freshly eclosed ergatoids, and may also grasp with their mandibles adult ergatoid rivals that somehow have survived the sensitive phase directly after emergence to daub them with hindgut secretions. Such besmearing elicits worker aggression, which results in the elimination of the contaminated male [6]. Survivors of these fights can mate with all female sexuals that eclose in their natal nests until they die or are killed by a younger rival [7,8]. Frequent mating is possible because, in contrast to males of all other social Hymenoptera, the testes of ergatoid *Cardiocondyla *males persist throughout their whole lives, resulting in a permanently replenishable sperm supply [9,10].

Winged *C. obscurior *males are larger and heavier and have weak mandibles, large eyes, and well-developed ocelli and wings. They are peaceful and represent a "disperser" morph, which appears to be produced predominantly under deteriorating environmental conditions [11]. In the first few days of their adult lives, winged males may attempt to mate with female nestmates. During this time they have fully functional testes and are protected against attacks by ergatoid male through chemically mimicking female sexuals [12]. However, a few days after adult emergence, spermatogenesis ceases, the sperm supply becomes limited, and winged males disperse to mate with female sexuals away from their natal nests.

Like in other male-polymorphic species, the two strikingly different reproductive tactics might arguably lead to different reproductive success ([13-15]; for additional examples see review [16]).

Based on life-long spermatogenesis in ergatoid males and the fact that they can monopolize a harem of female sexuals through lethal fighting, Anderson et al. [17] estimated that adult ergatoid males have a higher reproductive success than winged males. Conversely, from the number of observed copulation attempts, Tsuji et al. [18] concluded that the average fitness of adult winged males is higher. For a full understanding of the significance of male polymorphism in *Cardiocondyla*, more solid information on the reproductive success of the two alternative morphs is therefore needed.

In this study, we present data on the mating frequencies (number of inseminated females) and survival probability of ergatoid and winged males in *C. obscurior *and estimate their average mating success. Furthermore, because individuals in insect societies may have opposing interests about their own development (e.g., female caste conflict [19-21]), we also investigate whether the interests of male larvae concerning their own development coincide with those of the workers and the rest of the society.

## Results

We investigated the average mating success of ergatoid and winged males by determining,

a) directly through observations and dissections and indirectly through sperm counts, how many virgin female sexuals either male morph can inseminate during its life (reproductive potential),

b) the probability of an immature winged and ergatoid male surviving until sexual maturity (survival probability and longevity), and

c) the average number of rival males with which a given male has to share the female sexuals of its natal nest (mean number of competitors).

### Reproductive potential

**Figure 1:**
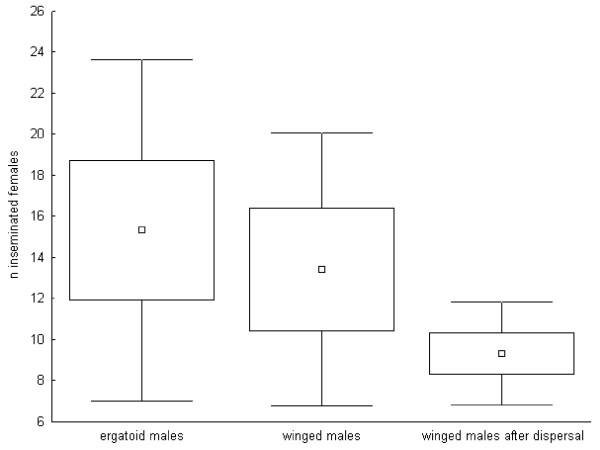
**Lifetime mating frequency of the different male morphs**. Lifetime mating frequency (number of inseminated female sexuals; mean, standard error and standard deviation) of ergatoid and winged males of the ant *Cardiocondyla obscurior*. The number of female sexuals available for each male was not limited, and colonies did not contain competing males.

The most successful ergatoid male inseminated 30 of 200 (15%) available female sexuals in its 19 day-long life, and the most active winged male inseminated 23 of 120 (19%) available females in 11 days. The individual lifetime mating frequencies differed neither between morphs nor between winged males allowed to remain in their natal nests and winged males that could mate only after attempting to disperse from their natal nest a few days after eclosion (ergatoid males: n = 6; min-max: 6 – 30; mean ± standard deviation: 15.3 ± 8.3; non-dispersing winged males: n = 5; min-max: 6 – 23; mean ± standard deviation: 13.4 ± 6.7; dispersing winged males: n = 6; min-max: 5 – 12; mean ± standard deviation: 9.3 ± 2.5; Anova: F = 1.42, P = 0.27; post-hoc LSD comparisons: all P > 0.1; Figure 1; power calculation: critical F = 3.68, power = 25%). Though the power of the statistical analysis is low, the large variation in the mating frequency among individual males suggests that morphs do not differ strongly in this respect.

**Figure 2:**
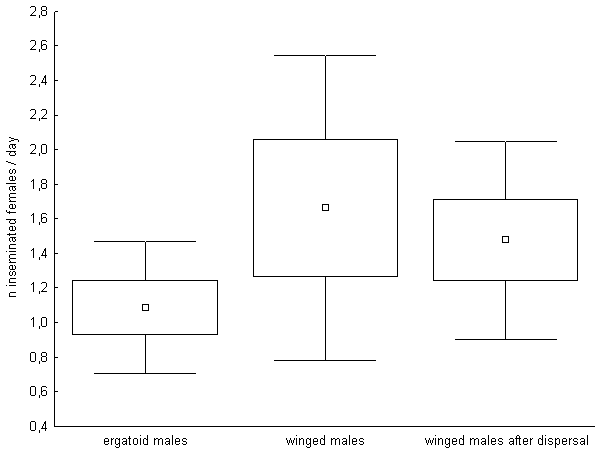
**Daily mating frequency of the different male morphs**. Daily mating frequency (number of inseminated female sexuals/day; mean, standard error and standard deviation) of ergatoid and winged males of the ant *Cardiocondyla obscurior*.

Similarly, the three types of males did not differ in their daily mating frequencies (ergatoid males: n = 6; min-max: 0.5 – 1.6; mean ± standard deviation: 1.1 ± 0.4; non-dispersing winged males: n = 5; min-max: 0.5 – 2.8; mean ± standard deviation: 1.7 ± 0.9; dispersing winged males: n = 6; min-max: 0.7 – 2.4; mean ± standard deviation: 1.5 ± 0.6; Anova: F = 1.23, P = 0.32; post-hoc LSD comparisons: all P > 0.3; Figure 2; power calculation: critical F = 3.68, power = 24%). However, daily dissections of females revealed that winged males inseminated on average two females per day during the first days (on average four days) of their life and later on one female per day, whereas ergatoid males consistently inseminated one female per day throughout their whole lives.

In our laboratory experiment, males were simultaneously provided with unrealistically large numbers of virgin females. In natural colonies, the number of female sexuals will be limited, and whereas the stationary ergatoid males cannot do much to increase their mating frequency, dispersing winged males may benefit from additional matings outside their natal nests. Comparing the sperm content of the seminal vesicles of adult winged males and of the spermatheca of inseminated queens allowed estimating for how many matings the limited sperm supply of a dispersing winged male would suffice. Sperm counts corroborated dissection data on the mating performance of winged males. After the completion of spermatogenesis, the two seminal vesicles of winged males contained on average a total of 22733 ± SD 5561 sperm cells (n = 8; 6204 ± SD 1134 sperm cells per count in a half of a seminal vesicle, n = 2 and 3; 2747 ± SD 832 sperm cells per count in a quarter of a seminal vesicle, n = 6 and 5).

**Figure 3:**
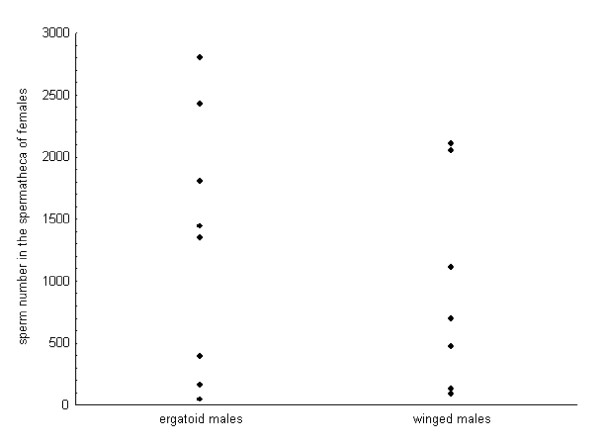
**Sperm number in the spermatheca of females**. Sperm number in the spermatheca of the females after one single copulation with the ergatoid or the winged male morph, respectively.

All seven female sexuals that were allowed to mate with a single winged male each were inseminated, although the amount of sperm cells in their spermatheca differed considerably (min-max: 91 – 2111; mean 953 ± SD 845; Figure 3). Assuming that no sperm is lost during copulation, the limited sperm supply of dispersing winged males would therefore be sufficient for 10 (maximal fill) to 24 (average fill) additional matings. This means that even when winged males had no chance of mating in their natal nest they would still be able to inseminate a considerable number of non-nestmate females. Indeed, dispersing winged males were observed to inseminate up to 12 female sexuals.

Spermathecae from three of 11 female sexuals allowed mating with ergatoid males did not contain any sperm, the others contained between 50 and 2796 spermatozoa (mean 1304 ± SD 1033; Figure 3). The amount of sperm transferred to the female spermathecae did not differ between winged and ergatoid males (n_1 _= 7, n_2 _= 8; t = 0.71, P = 0.49, power calculation: critical value of t: 2.16, power = 10%), and was also not correlated with the duration of copulation (Spearman Rank correlation: ergatoid males, n = 8, r_S _= 0.17, p = 0.69; winged males, n = 7, r_S _= 0.22, p = 0.64).

Considering that *C. obscurior *queens have a mean lifespan of 26 weeks [22] and lay on average two eggs per day, 400 spermatozoa are sufficient to fertilize all eggs laid throughout their whole lives.

### Survival probability, longevity, and mean number of competitors

Only nine of 71 ergatoid male pupae, whose fate was observed during this study, survived to adulthood (12.7%), and most were killed by adult rivals (54 of 71, 76.1%). In eight cases, both the young and the old males were killed by workers after besmearing each other with hindgut content (11.3%). Of 51 observed adult ergatoid males, five (9.8%) died without being involved in any male-male interaction, 29 killed one to six younger rivals (mean 1.86 ± 1.36; 56.9%), nine (17.6%) were replaced in a fight by a younger male and eight (15.7%) were killed by workers after fighting with a younger ergatoid male (see above). Thus, an ergatoid male larva has an average chance of surviving to adulthood of 13%, while adult ergatoid males have a 2/3 chance of surviving the eclosion of young rivals. Ergatoid males that were killed by younger rivals lived on average for 12.5 ± SD 6.4 days and had thus a considerable chance of mating multiply before death. All together, ergatoid males lived on average as long as winged males once they had survived the critical first few hours after adult emergence (mean winged males: 12.25 ± 5.75; mean ergatoid males: 17.43 ± 14.08; Cox-Mantel-test: n_1 _= 12, n_2 _= 51, U = 3.55, p = 0.16), and some ergatoid males reached a much higher maximum lifespan than winged males (60 vs. 28 days).

Under normal environmental conditions, most colonies do not contain winged males and adult ergatoid males can thus monopolise mating with all female sexuals, i.e., under normal conditions their probability of mating with a given female sexual in their nest is 1. This is different for winged males, because 24 of 53 (45.3%) colonies in which they were produced contained multiple males (in six colonies one ergatoid and one winged male each, in two colonies one ergatoid and two winged males, in 16 colonies two to five winged males; averaged over all 53 colonies 1.45 ± 0.69 males). If female sexuals mated singly and all nestmate males had equal access to females, winged males would thus have a probability of mating with a given female nestmate of 0.77. For the more likely case of multiple mating see below.

### Estimation of the mean and maximum reproductive success of the different male morphs

**Figure 4:**
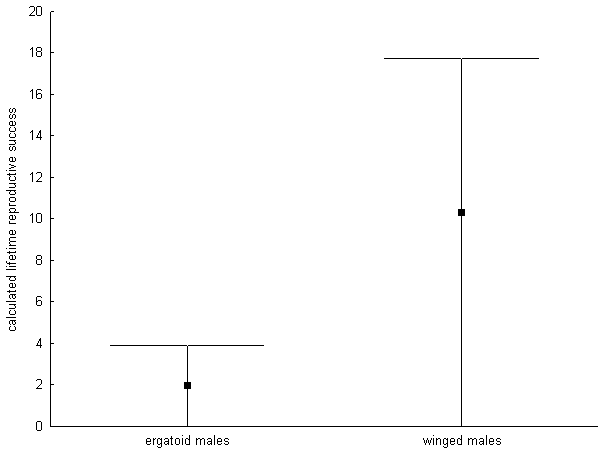
**Calculated lifetime mating success of the different male morphs**. Mean, minimum (always zero) and maximum calculated lifetime mating success (lifetime matings * survival probability * probability of mating) of ergatoid and winged males of the ant *Cardiocondyla obscurior*.

The observed average lifetime mating frequency did not differ between winged and wingless males (see above, Figure 1) and thus appears to be unaffected by the degeneration of testes in winged males. However, exceptional ergatoid males may inseminate many more female sexuals than winged males due to their unlimited sperm supply. In contrast to ergatoid males, winged males do not experience increased mortality before or shortly after adult eclosion. In colonies with multiple winged males or with a winged and an ergatoid male, males have to share mating opportunities with competitors. For a crude estimate of male mating success allowing for the presence of multiple males per colony, we here assume that most adult ergatoid males do not have to compete for female sexuals and that all winged male pupae reach adulthood. The average lifetime mating success as well as the estimated maximum lifetime mating success of winged males is then considerably larger than that of ergatoid males (mean number of lifetime matings * survival probability * probability of mating: ergatoid males: 15.3 * 0.13 * 1 = 1.99; winged males: 13.4 * 1 * 0.77 = 10.3; maximum number of lifetime matings * survival probability * probability of mating: ergatoid males: 30 * 0.13 *1 = 3.9; winged males: 23 * 1 * 0.77: 17.71; Figure 4).

If queens mated multiply, the presence of rival males could be ignored and both ergatoid and winged males would have an equal probability of mating of 1. In this case, the difference between winged and ergatoid males would be larger, although the reproductive output of each individual male would be reduced due to shared paternities. Similarly, if the number of female sexuals in the natal nest were limited, winged males would have a higher chance of mating because of their capability of mating away from the nest. Hence, male larvae should prefer to grow to winged adults if they were in control of their own development.

## Discussion

Ergatoid and winged males of *Cardiocondyla obscurior *appear to have largely overlapping lifetime mating frequencies, though a few ergatoid males because of their longer lifespan and replenishable sperm supply may eventually reach higher mating frequencies than winged males. As both male morphs transfer on average similar amounts of sperm during copulation, equal mating frequency translates into equal fitness of mature males. However, because most (7 of 8) immature ergatoid males are killed by their adult rivals, their average fitness is much lower than that of winged males. A male larva is therefore expected to prefer developing into a winged adult. Though the limited sample sizes in our analyses resulted in a rather low statistical power, our conclusion would presumably hold even if ergatoid males had consistently higher mating frequencies because of their much lower probability of surviving till adulthood.

Fitness differences might be even more pronounced under more natural conditions than in our laboratory experiments. For example, in natural colonies the number of female sexuals is much more limited and varies with season. In our study population in Bahia, Brazil, female sexual production peaked in April [23], whereas in Okinawa, Japan, female sexuals of this or a closely related species were found mostly from May till August [24]. Even in the laboratory, in 25% of those colonies in which we investigated the survival probability of ergatoid males, not a single female sexual eclosed during the whole lifetime of the male. Restricted availability of nestmate females strongly reduces the average mating success of ergatoid males but probably affects less the mating chances of winged males. They can react flexibly to the conditions in the nest by mating with the female sexuals present and later leaving with a sperm supply still sufficient to inseminate several female sexuals outside of the nest. Observations suggest that winged males indeed time the dispersal from their natal nests based on the availability of female sexuals (S. Cremer, unpubl.; own observation).

Furthermore, we have assumed that winged males usually have to share mating with female sexuals with other males, because they are not able of monopolizing mating chances. According to genetic data, queens of other *Cardiocondyla *species mate multiply ([25,26], Schrempf and Heinze, unpubl.), and *C. obscurior *queens have also been observed to mate with several males in the laboratory. If queens mated with all males present in the colony and the sperm of different males would be used equally, the co-occurrence of multiple males would not affect a male's mating frequencies, and the fitness advantage for winged males would remain.

Nevertheless, most natural colonies only contain ergatoid males [17,23], and winged males appear to be produced only under certain environmental conditions, such as a sudden temperature decrease or colony fragmentation [11]. Why don't male larvae pursue their fitness interests and develop into winged males?

The answer is probably simple: similar to caste differentiation in female larvae of most social Hymenoptera, male larvae lack the power to enforce their interests, because their development is completely controlled by the workers [27]. Workers of *C. obscurior *react to a sudden temperature drop by more frequently biting and antennating male larvae and in this way in late 2^nd ^instar larvae switch on the pathway leading to winged males. A similar behaviour has been reported for *Myrmica *ants, where workers through biting prevent female larvae from developing into queens and force them to develop into workers [28]. After experiencing a temperature decrease, workers of *C. obscurior *reared winged males even from larvae that had not been subjected to stressful conditions themselves [29].

Under standard conditions, the optimal allocation strategy of workers appears to be rearing exclusively ergatoid males. Ergatoid males are smaller and lighter [11], develop faster than winged males [29] and are therefore produced at lower costs, which can be further reduced through "recycling:" ergatoid males that have been killed by rivals are fed to the larvae, so that investment in surplus ergatoid males is not completely lost. Under normal conditions, a single ergatoid male suffices for inseminating all female sexuals present in a colony. Rearing cheap, ergatoid males, which are either killed and recycled or may replace an adult ergatoid in the case of its death, obviously meets the inclusive fitness interests of workers more than producing expensive winged males, which would compete with the adult ergatoid male for the limited mating chances.

Our research might thus reveal a novel type of potential kin conflict in insect societies – between individual male larvae and the colony. The outcome of male-worker conflict in *C. obscurior *appears to be similar to that of the conflict about caste fate. With rare exceptions [30-32], workers appear to have the power to enforce their interests because they control the investment into larvae, e.g., though providing the appropriate quality or quantity of nutrition [19-21]. In *Cardiocondyla*, both caste and male dimorphism appear to be based on similar proximate mechanisms [29,33], and workers can probably prevent male larvae from developing to winged sexuals simply by limiting their food intake. Though sociality offers pluripotent larvae unique opportunities of manipulating their own rearing conditions, e. g., through more intensively begging for food [30] or signalling hunger [34], the alternative male tactics in *C. obscurior *appear to be the result of a conditional strategy controlled by the workers, which allows the latter to maximise their inclusive fitness.

## Conclusion

Winged males appear to have a much higher average mating success than ergatoid males, because most of the latter are killed before reaching maturity. Nevertheless, under standard environmental conditions the optimal allocation strategy of workers is to rear exclusively the less costly ergatoid males. This results in a potential conflict between male larvae and workers, which, however, does not lead to overt conflict, as workers are in complete control of male larval development.

## Methods

### Study species

Colonies of *C. obscurior *were collected from their nests in rolled leaves in experimental lemon plantations of CEPLAC at Ilhéus and Una, Bahia, Brazil. In the laboratory, ants were kept under near-natural conditions in climatic chambers with 30°C/25°C temperature and 12 h/12 h day/night-cycles (for details see [9]). While ergatoid males and female sexuals are produced year-round, winged male production was initiated by splitting large colonies into smaller subunits (see e.g. [11]).

### Reproductive potential

To estimate the maximum and mean mating frequency of males, we placed 1 day old ergatoid (n = 6) and winged males (n = 5) individually in nest chambers with ten to 20 female sexuals, approximately 15 workers, and some brood each. Ten female sexuals from several large "donor" colonies were added per day to each of these set-ups until the male died. We repeated the experiment with virgin winged males after these had apparently attempted to disperse from their natal nest a few days after eclosion (on average after mean ± SD: 6.0 ± 2.8 days), to detect whether there are differences in mating frequencies between the two groups of winged males, as in the latter, sperm supply is limited (due to testes degeneration after a few days of adulthood, see above). All female sexuals (n = 1870) were later dissected to determine whether their spermathecae contained sperm or not. The availability of female sexuals limited the number of males for which mating frequency could be determined.

For sperm counts, 8 – 10 d old winged males (n = 8) without prior sexual experience were dissected on a microscope slide in a drop of Beadle solution (128.3 mM NaCl, 4.7 mM KCl, 2.3 mM CaCl_2_; [35]). Both seminal vesicles were transferred into a new drop of Beadle solution each and sperm was released and mixed carefully with the tip of the forceps to avoid clumping. The position of the sperm mass was marked in ink on the bottom of the slide and the solution was allowed to evaporate. Sperm was then fixed in 70% ethanol and stained with DAPI (Hoechst; [36]). The two droplets were divided into quarters, and two persons separately counted the number of sperm present in one quarter (n = 5 and 6) or half (n = 3 and 2) of a seminal vesicle of each male using a fluorescence microscope at a magnification of ×200. After control by eye (microscope, magnification ×200) that sperm of both seminal vesicles had been mixed carefully and did not cluster in one part of the droplets, the mean number of total sperm was estimated from the counts. There was no significant difference in sperm counts between the two observers (t-test: t = -0.42, P > 0.6; mean difference between the counts: 235 (half vesicle) and 184 sperm cells (quarter vesicle), i.e., counting errors were approximately 4% and 7%, respectively).

Female sexuals (n = 18) were allowed to mate with a single male each that had not copulated previously (11 ergatoid males and seven winged males). All copulations were observed by eye and videotaped and their durations were recorded. Immediately after the copulation, we separated the male from the female sexual to avoid repeated mating among the same partners. The spermathecae of young queens were dissected in a drop of Beadle solution 24 hours after the copulation and all sperm in the drop was counted as above by two separate persons (mean difference between two counts: 80 (mated with winged males) and 53 (mated with ergatoid males) sperm cells; i.e., approximately 6% and 4% counting error; t-test: t = 0.66, P > 0.5). We expected that sperm transferred during the copulation had by this time completely migrated form the bursa copulatrix into the queen's spermatheca.

### Survival probability, longevity, and mean number of competitors

The mean adult lifespan of males was determined by marking freshly eclosed males in their colonies, from which adult ergatoid males had previously been removed, to prevent that the eclosing male was killed (51 males, 47 colonies). Afterwards, the number and coloration of male pupae (young, white vs. brown pupae ready to eclose, n = 71) were recorded four times per week. The number of dead old males and killed, freshly eclosed ergatoid males was recorded to estimate the life span of adult males and the probability of ergatoid male pupae reaching reproductive life.

To investigate how many males compete on average for female sexuals in a single colony, we recorded the mean number of males in 53 colonies that produced winged males over a period of four weeks. In colonies, in which only ergatoid males were produced, one adult male monopolized all matings.

## Authors' contributions

ED introduced AS into the technique of sperm staining and took part in sperm counts. AS carried out the remaining experiments. JH supervised the study, and AS and JH wrote the ms. All authors read and approved the final manuscript.
